# The omics era: a nexus of untapped potential for Mendelian chromatinopathies

**DOI:** 10.1007/s00439-023-02560-2

**Published:** 2023-04-28

**Authors:** Aileen A. Nava, Valerie A. Arboleda

**Affiliations:** 1 Department of Human Genetics, David Geffen School of Medicine, UCLA, Los Angeles, CA USA; 2 Department of Pathology & Laboratory Medicine, David Geffen School of Medicine, UCLA, Los Angeles, CA USA; 3 Department of Computational Medicine, David Geffen School of Medicine, UCLA, Los Angeles, CA USA; 4 Broad Stem Cell Research Center, University of California, Los Angeles, CA USA; 5 Molecular Biology Institute, University of California, Los Angeles, CA USA; 6 Jonsson Comprehensive Cancer Center, University of California, Los Angeles, CA USA

## Abstract

The OMICs cascade describes the hierarchical flow of information through biological systems. The epigenome sits at the apex of the cascade, thereby regulating the RNA and protein expression of the human genome and governs cellular identity and function. Genes that regulate the epigenome, termed epigenes, orchestrate complex biological signaling programs that drive human development. The broad expression patterns of epigenes during human development mean that pathogenic germline mutations in epigenes can lead to clinically significant multi-system malformations, developmental delay, intellectual disabilities, and stem cell dysfunction. In this review, we refer to germline developmental disorders caused by epigene mutation as “chromatinopathies”. We curated the largest number of human chromatinopathies to date and our expanded approach more than doubled the number of established chromatinopathies to 179 disorders caused by 148 epigenes. Our study revealed that 20.6% (148/720) of epigenes cause at least one chromatinopathy. In this review, we highlight key examples in which OMICs approaches have been applied to chromatinopathy patient biospecimens to identify underlying disease pathogenesis. The rapidly evolving OMICs technologies that couple molecular biology with high-throughput sequencing or proteomics allow us to dissect out the causal mechanisms driving temporal-, cellular-, and tissue-specific expression. Using the full repertoire of data generated by the OMICs cascade to study chromatinopathies will provide invaluable insight into the developmental impact of these epigenes and point toward future precision targets for these rare disorders.

## Introduction

The human body is made of trillions of cells and hundreds of unique cell types, which arose from a single cell. The nucleus of the primordial single cell: the zygote, includes two sets of instructions that guide human development: the genome and the epigenome. The genome (i.e., deoxyribonucleic acid; DNA) remains constant across all cells in an organism while the epigenome varies between cells and directs cell-type specification by controlling DNA organization through chemical modifications (Deans and Maggert 2015). Each human possesses thousands of cell-type-specific epigenomes (Moss et al. 2018; Horvath 2013; Mo et al. 2015) that are inherited during cell division (Lacal and Ventura 2018).

This review covers core concepts in gene regulation, epigenomics, and human disease. We collectively refer to human developmental disorders caused by germline mutations in genes that control epigenome function as “chromatinopathies''. Each chromatinopathy is considered a rare disorder, affecting fewer than 200,000 people in the United States (Hoskins 2022). First, we define the epigenome and use large-scale data to expand the number of monogenic disorders defined as chromatinopathy syndromes. Previous reviews have restricted the definition of chromatinopathies to neurodevelopmental disorders caused by pathogenic mutations in canonical chromatin-modifier or chromatin-remodeler genes (Berdasco and Esteller 2013; Bjornsson 2015; Fahrner and Bjornsson 2019; Van Gils et al. 2021; Luperchio et al. 2021). The second major focus in the review is on -OMICs technology which is used to elucidate causal mechanisms driving these rare and severe developmental disorders. We review established and emerging molecular technologies designed to assess layers in the -OMICs cascade and how these assays have been implemented to investigate the pathogenesis and pathophysiology of select chromatinopathies.

## Defining the epigenome

**Fig. 1:**
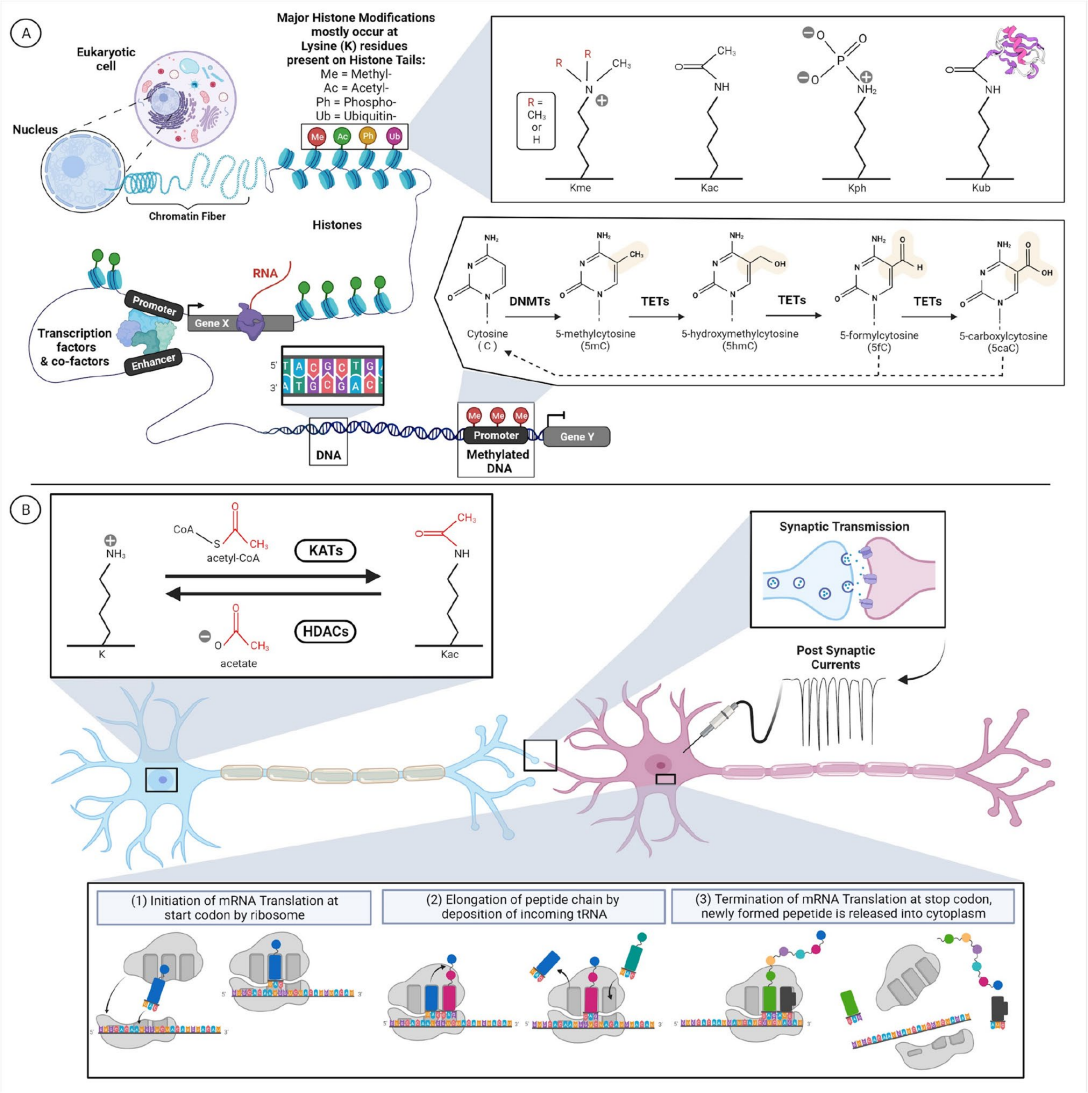
Visualization of omics layers in biological systems. **A** Snapshot of gene regulation in Eukaryotic cell by the epigenome and genome. DNA Methyltransferases (DNMTs) write/deposit DNA methylation, while ten-eleven translocation (TETs) enzymes erase/remove methyl-groups from DNA. While different classes of enzymes write/deposit, erase/remove, and maintain the 4 major histone post-translational modifications shown above. **B** Snapshot of neurons communicating to product a cellular phenotype that can be assessed through electrophysiology to measure rate of synaptic transmission. Synaptic transmission can be driven by changes in histone acetylation, which is a metabolic reaction mediated by genes encoding lysine (K) acetyltransferases (KATs) and histone deacetylases  (HDACs) to cause changes in gene expression which translates to changes in protein abundance within neurons

The epigenome was originally defined as the study of heritable changes in gene expression and function which do not alter the DNA (Wu and Morris 2001). In Fig. 1, a key function of the epigenome is to regulate the three-dimensional (3D) organization of chromatin to partition the genome such that only a fraction of genomic DNA is physically accessible to biological machinery for transcription into ribonucleic acid (RNA). In combination with the human genome, this enables the epigenome to control the spatial and temporal timing of gene expression in a cell-specific manner. There are five major chemical modifications present on chromatin that influence the cell’s epigenetic state: DNA methylation, histone methylation, histone acetylation, histone phosphorylation, and histone ubiquitination as well as dozens of low-abundance chemical modifications (Ludwig and Bintu 2019). In this review, we define ‘epigenes’ as genes encoding proteins that affect a cell's epigenome (Sadakierska-Chudy et al. 2015; Medvedeva et al. 2015). These epigenes can be divided into four groups: (1) ‘chromatin-modifiers’ are proteins that interact and/or regulate histone post-translational modifications, (2) ‘chromatin remodelers’ are proteins that regulate the structure/organization of chromatin, (3) proteins that modulate chemical modification present on DNA/RNA, and lastly (4) accessory proteins that are essential in epigenome-altering processes (Sadakierska-Chudy et al. 2015; Sadakierska-Chudy and Filip 2015; Javaid and Choi 2017) and their functions are reviewed in Medvedeva et al.

In addition to transcriptomic regulation through histone post-translational modifications, transcript expression can be regulated through direct post-transcriptional modifications to nucleic acids (i.e., DNA/RNA). For example, some epigenes modulate the presence of chemical modifications on messenger (mRNA) and regulate their stability within a cell, thereby influencing gene expression (Chen et al. 2016, 2020; Roundtree et al. 2017). However, significantly fewer chemical modifications are known to exist on DNA/RNA as compared to histones (Ludwig and Bintu 2019). Nucleic acid methylation occurring at cytosine or adenine nucleotides is the most abundant and best studied epigenetic chemical modification on DNA. However the proteins responsible for writing, erasing, or reading RNA methylation are still being identified (Boo and Kim 2020). The last group of epigenes indirectly function in epigenome-altering processes by serving as chaperones, scaffolds, or cofactors (Medvedeva et al. 2015). Thus, these epigenes collectively regulate an organism’s epigenome through a multitude of biological and molecular processes. While the epigene definition here does not explicitly include non-coding RNAs (ncRNAs), it is important to highlight that ncRNAs are critical gene regulatory elements that regulate fundamental processes, such as X-inactivation (Engreitz et al. 2013; Chitiashvili et al. 2020), and are reviewed in (Beermann et al. 2016). This review primarily focuses on genetic syndromes caused by germline mutations in protein-coding epigenes (Berdasco and Esteller 2013)**.**

## Expanding the chromatinopathy landscape through data mining

A comprehensive study of epigenetic factors identified 720 epigenes after filtering out 95 genes that encode histones and protamines (Medvedeva et al. 2015). To illuminate the extent to which pathogenic epigene mutations cause monogenic developmental disorders, a.k.a chromatinopathies, we filtered 720 epigenes against the largest publicly-available human geno-phenotype database: Online Mendelian Inheritance in Man (OMIM) (Hamosh et al. 2005; Amberger et al. 2015). We found that 29.6% (213/720) of epigenes are associated with at least one human morbidity. We identified these genes by mapping their HGNC IDs to OMIM’s morbid accession IDs using the ensembl database for human genes (GRCh38.p13; downloaded June 2022 through the R package biomaRt (Smedley et al. 2009). Collectively, these 213 unique epigenes are mapped to 322 OMIM morbid accession IDs, resulting in a list of 322 genotype–phenotype pairs that contained repeated elements due to the polygenic-nature of some OMIM phenotypes and the pleiotropic-nature of some epigenes.

Therefore, to generate a high-confidence list of chromatinopathies shown in Table 1, we then filtered these 322 genotype–phenotype pairs to remove entries that were not monogenic developmental disorders/syndromes. Specifically, genotype–phenotype entries were removed if the OMIM phenotype: (1) did not have a clear mode of inheritance, (2) was caused by somatic mutations, or (3) was not a syndromic developmental disorder. After filtering these 322 genotype–phenotype pairs, we found that 20.6% (148/720) of all epigenes cause at least one chromatinopathy (Table 1). Specifically, we identified 179 chromatinopathies that are caused by pathogenic germline mutations in 148 distinct epigenes using our data-mining strategy. This doubles previous estimates, which report 40–70 chromatinopathy-causing epigenes (Fahrner and Bjornsson 2019; Valencia and Pașca 2022). The ability to expand the current Chromatinopathy landscape, which we extensively cataloged in Table 1, is fueled by two evolving sources of information: (1) the continuous identification of novel genes associated with genetic syndromes and (2) the elucidation of the mechanistic basis underlying a protein’s capacity to influence gene regulation through the epigenome. Accordingly, we expect the proportion of chromatinopathy-causing epigenes will increase as more children are diagnosed using state-of-the-art genome sequencing technologies.

**Table 1:** Summary of 179 Chromatinopathies caused by germline mutations in 148 epigenes

HGNC ID:	HGNC Symbol:	Epigenome Function:	OMIM ID:	Inheritance:	Condition:
132	ACTB	Chromatin remodeling cofactor	243,310	AD	BARAITSER–WINTER SYNDROME 1; BRWS1. (Alternative name: FRYNS–AFTIMOS SYNDROME)
160	ACTL6B	Chromatin remodeling cofactor	618,468	AR	DEVELOPMENTAL AND EPILEPTIC ENCEPHALOPATHY 76; DEE76
160	ACTL6B	Chromatin remodeling cofactor	618,470	AD	INTELLECTUAL DEVELOPMENTAL DISORDER WITH SEVERE SPEECH AND AMBULATION DEFECTS; IDDSSAD
15,766	ADNP	Chromatin remodeling cofactor	615,873	AD	HELSMOORTEL–VAN DER AA SYNDROME; HVDAS
13,203	AICDA	DNA modification	605,258	AR	HYPER-IgM SYNDROME 2
360	AIRE	Histone modification read, TF	240,300	AD, AR	AUTOIMMUNE POLYENDOCRINE SYNDROME, TYPE I, WITH OR WITHOUT REVERSIBLE METAPHYSEAL DYSPLASIA; APS1
11,110	ARID1A	Chromatin remodeling cofactor	614,607	AD	COFFIN–SIRIS SYNDROME 2; CSS2
18,040	ARID1B	Histone modification write	135,900	AD	COFFIN–SIRIS SYNDROME 1; CSS1
18,037	ARID2	Chromatin remodeling cofactor	617,808	AD	COFFIN–SIRIS SYNDROME 6; CSS6
19,088	ASH1L	Histone modification write	617,796	AD	MENTAL RETARDATION AUTOSOMAL DOMINANT 52; MRD52
18,318	ASXL1	Histone modification erase, Polycomb group (PcG) protein	605,039	AD	BOHRING–OPITZ SYNDROME; BOS
23,805	ASXL2	Histone modification read	617,190	AD	SHASHI–PENA SYNDROME; SHAPNS
29,357	ASXL3	Scaffold protein, Polycomb group (PcG) protein	615,485	AD	BAINBRIDGE–ROPERS SYNDROME; BRPS
795	ATM	Histone modification write	208,900	AR	LOUIS–BAR SYNDROME
3033	ATN1	Histone modification erase cofactor	125,370	AD	HAW RIVER SYNDROME; HRS
882	ATR	Histone modification write	210,600	AR	SECKEL SYNDROME 1; SCKL1
882	ATR	Histone modification write	614,564	AD	CUTANEOUS TELANGIECTASIA AND CANCER SYNDROME, FAMILIAL; FCTCS
886	ATRX	Chromatin remodeling	301,040	XLD	ALPHA-THALASSEMIA/MENTAL RETARDATION SYNDROME, X-LINKED; ATRX
886	ATRX	Chromatin remodeling	309,580	XLR	MENTAL RETARDATION-HYPOTONIC FACIES SYNDROME, X-LINKED, 1; MRXHF1. (Alternative names: SMITH–FINEMAN–MYERS SYNDROME 1, CARPENTER–WAZIRI SYNDROME, CHUDLEY–LOWRY SYNDROME, HOLMES–GANG SYNDROME)
950	BAP1	Histone modification erase, Polycomb group (PcG) protein	614,327	AD	TUMOR PREDISPOSITION SYNDROME; TPDS
20,893	BCOR	Polycomb group (PcG) protein	300,166	XLD	MICROPHTHALMIA, SYNDROMIC 2; MCOPS2
25,657	BCORL1	Histone modification erase cofactor	301,029	XLR	SHUKLA–VERNON SYNDROME; SHUVER
3581	BPTF	Chromatin remodeling	617,755	AD	NEURODEVELOPMENTAL DISORDER WITH DYSMORPHIC FACIES AND DISTAL LIMB ANOMALIES; NEDDFL
1100	BRCA1	Histone modification write cofactor, TF	617,883	AR	FANCONI ANEMIA, COMPLEMENTATION GROUP S; FANCS
1101	BRCA2	Histone modification write	605,724	AR	FANCONI ANEMIA, COMPLEMENTATION GROUP D1; FANCD1;;FAD1
14,255	BRPF1	Histone modification read	617,333	AD	INTELLECTUAL DEVELOPMENTAL DISORDER WITH DYSMORPHIC FACIES AND PTOSIS; IDDDFP
17,342	BRWD3	Histone modification read	300,659	XLR	X-LINKED INTELLECTUAL DEVELOPMENTAL DISORDER 93; XLID93
1744	CDC6	Chromatin remodeling	613,805	AR	MEIER-GORLIN SYNDROME 5; MGORS5
16,783	CDC73	Histone modification write cofactor	145,001	AD	HYPERPARATHYROIDISM-JAW TUMOR SYNDROME; HPT-JT
1915	CHD1	Chromatin remodeling	617,682	AD	PILAROWSKI–BJORNSSON SYNDROME; PILBOS
1917	CHD2	Chromatin remodeling	615,369	AD	DEVELOPMENTAL AND EPILEPTIC ENCEPHALOPATHY 94; DEE94
1918	CHD3	Chromatin remodeling	618,205	AD	SNIJDERS BLOK–CAMPEAU SYNDROME; SNIBCPS
1919	CHD4	Chromatin remodeling	617,159	AD	SIFRIM–HITZ–WEISS SYNDROME; SIHIWES
20,626	CHD7	Chromatin remodeling	214,800	AD	CHARGE SYNDROME
20,626	CHD7	Chromatin remodeling	612,370	AD	HYPOGONADOTROPIC HYPOGONADISM 5 WITH OR WITHOUT ANOSMIA; HH5
1974	CHUK	Histone modification write	613,630	AR	FETAL ENCASEMENT SYNDROME
1974	CHUK	Histone modification write	619,339	AR	BARTSOCAS–PAPAS SYNDROME 2; BPS2
18,688	CRB2	Histone modification read	219,730	AR	VENTRICULOMEGALY WITH CYSTIC KIDNEY DISEASE; VMCKD. (Alternative name: genetic steroid-resistant nephrotic syndrome)
18,688	CRB2	Histone modification read	616,220	AR	FOCAL SEGMENTAL GLOMERULOSCLEROSIS 9; FSGS9. (Alternative name: genetic steroid-resistant nephrotic syndrome)
2348	CREBBP	Histone modification write	180,849	AD	RUBINSTEIN–TAYBI SYNDROME 1; RSTS1
2348	CREBBP	Histone modification write	618,332	AD	MENKE–HENNEKAM SYNDROME 1; MKHK1
2457	CSNK2A1	Histone modification	617,062	AD	OKUR–CHUNG NEURODEVELOPMENTAL SYNDROME; OCNDS
2494	CTBP1	Chromatin remodeling	194,190	AD	WOLF–HIRSCHHORN SYNDROME; WHS
2494	CTBP1	Chromatin remodeling	617,915	AD	HYPOTONIA, ATAXIA, DEVELOPMENTAL DELAY, AND TOOTH ENAMEL DEFECT SYNDROME; HADDTS
13,723	CTCF	Chromatin remodeling, TF	615,502	AD	MENTAL RETARDATION AUTOSOMAL DOMINANT 21; MRD21
2553	CUL3	Histone modification write	619,239	AD	NEURODEVELOPMENTAL DISORDER WITH OR WITHOUT AUTISM OR SEIZURES; NEDAUS
2555	CUL4B	Histone modification write	300,354	XLR	MENTAL RETARDATION, X-LINKED, SYNDROMIC, CABEZAS TYPE; MRXSC. (Alternative name: CABEZAS SYNDROME)
2717	DDB1	Histone modification write	619,426	AD	WHITE–KERNOHAN SYNDROME; WHIKERS
2976	DNMT1	DNA modification	604,121	AD	CEREBELLAR ATAXIA, DEAFNESS, AND NARCOLEPSY, AUTOSOMAL DOMINANT; ADCADN
2976	DNMT1	DNA modification	614,116	AD	NEUROPATHY, HEREDITARY SENSORY, TYPE IE; HSN1E
2978	DNMT3A	DNA modification	615,879	AD	TATTON–BROWN–RAHMAN SYNDROME; TBRS
2978	DNMT3A	DNA modification	618,724	AD	HEYN–SPROUL–JACKSON SYNDROME; HESJAS
2979	DNMT3B	DNA modification	242,860	AR	IMMUNODEFICIENCY-CENTROMERIC INSTABILITY-FACIAL ANOMALIES SYNDROME 1; ICF1
9964	DPF2	Chromatin remodeling	618,027	AD	COFFIN–SIRIS SYNDROME 7; CSS7
3188	EED	Polycomb group (PcG) protein	617,561	AD	COHEN–GIBSON SYNDROME; COGIS
24,650	EHMT1	Histone modification write	610,253	AD	KLEEFSTRA SYNDROME 1; KLEFS1
5959	ELP1	Scaffold protein	223,900	AR	NEUROPATHY, HEREDITARY SENSORY AND AUTONOMIC, TYPE III; HSAN3. (Alternative name: RILEY–DAY SYNDROME)
3373	EP300	Histone modification write	613,684	AD	RUBINSTEIN–TAYBI SYNDROME 2; RSTS2
3373	EP300	Histone modification write	618,333	AD	MENKE–HENNEKAM SYNDROME 2; MKHK2
3438	ERCC6	Chromatin remodeling	133,540	AR	COCKAYNE SYNDROME B; CSB
3438	ERCC6	Chromatin remodeling	214,150	AR	CEREBROOCULOFACIOSKELETAL SYNDROME 1; COFS1
3438	ERCC6	Chromatin remodeling	278,800	AR	DE SANCTIS–CACCHIONE SYNDROME
3438	ERCC6	Chromatin remodeling	600,630	AR	UV-SENSITIVE SYNDROME 1; UVSS1
17,097	EXOSC2	Scaffold protein, RNA modification	617,763	AR	Retinitis pigmentosa-hearing loss-premature aging-short stature-facial dysmorphism syndrome
3519	EYA1	Histone modification erase	113,650	AD	BRANCHIOOTORENAL SYNDROME 1; BOR1. (Alternative name: MELNICK–FRASER SYNDROME)
3519	EYA1	Histone modification erase	166,780	AD	OTOFACIOCERVICAL SYNDROME 1; OTFCS
3519	EYA1	Histone modification erase	602,588	AD	BRANCHIOOTIC SYNDROME 1; BOS1
3527	EZH2	Histone modification write, Polycomb group (PcG) protein	277,590	AD	WEAVER SYNDROME; WVS
3823	FOXP1	Recruits specific chromatin-modifying complexes with HDAC activity, TF	613,670	AD	MENTAL RETARDATION WITH LANGUAGE IMPAIRMENT AND WITH OR WITHOUT AUTISTIC FEATURES
13,875	FOXP2	Recruits specific chromatin-modifying complexes with HDAC activity, TF	602,081	AD	SPEECH AND LANGUAGE DISORDER WITH OROFACIAL DYSPRAXIA
6106	FOXP3	Recruits specific chromatin-modifying complexes with HDAC activity, TF	304,790	XLR	IMMUNODYSREGULATION, POLYENDOCRINOPATHY, AND ENTEROPATHY, X-LINKED syndrome; IPEX
30,778	GATAD2B	Histone modification read	615,074	AD	GAND SYNDROME; GAND
4839	HCFC1	Chromatin remodeling	309,541	XLR	MENTAL RETARDATION, X-LINKED 3; MRX3
14,063	HDAC4	Histone modification erase	600,430	AD	CHROMOSOME 2q37 DELETION SYNDROME. (Alternative name: BRACHYDACTYLY-MENTAL RETARDATION SYNDROME)
13,315	HDAC8	Histone modification erase	300,882	XLD	CORNELIA DE LANGE SYNDROME 5; CDLS5
4861	HELLS	Chromatin remodeling	616,911	AR	IMMUNODEFICIENCY-CENTROMERIC INSTABILITY-FACIAL ANOMALIES SYNDROME 4; ICF4
30,892	HUWE1	Histone modification write	309,590	XLD, XLR	MENTAL RETARDATION, X-LINKED, SYNDROMIC, TURNER TYPE; MRXST. (Alternative names: JUBERG–MARSIDI SYNDROME, BROOKS–WISNIEWSKI–BROWN SYNDROME)
24,565	KANSL1	Histone modification write cofactor	610,443	AD	KOOLEN–DE VRIES SYNDROME; KDVS
5275	KAT5	Histone modification write	619,103	AD	NEURODEVELOPMENTAL DISORDER WITH DYSMORPHIC FACIES, SLEEP DISTURBANCE, AND BRAIN ABNORMALITIES; NEDFASB
13,013	KAT6A	Histone modification write	616,268	AD	ARBOLEDA–THAM SYNDROME; ARTHS. (Alternative name: KAT6A SYNDROME)
17,582	KAT6B	Histone modification write	603,736	AD	SAY–BARBER–BIESECKER–YOUNG–SIMPSON SYNDROME; SBBYSS. (Alternative name: OHDO SYNDROME, SBBYS VARIANT)
17,582	KAT6B	Histone modification write	606,170	AD	GENITOPATELLAR SYNDROME; GTPTS
17,933	KAT8	Histone modification write	618,974	AD	LI–GHORBANI–WEISZ–HUBSHMAN SYNDROME; LIGOWS
29,079	KDM1A	Histone modification erase	616,728	AD	CLEFT PALATE, PSYCHOMOTOR RETARDATION, AND DISTINCTIVE FACIAL FEATURES; CPRF
1337	KDM3B	Histone modification erase	618,846	AD	DIETS–JONGMANS SYNDROME; DIJOS
29,136	KDM4B	Histone modification erase	619,320	AD	MENTAL RETARDATION AUTOSOMAL DOMINANT 65, MRD65
18,039	KDM5B	Histone modification erase	618,109	AR	MENTAL RETARDATION AUTOSOMAL RECESSIVE 65; MRT65
11,114	KDM5C	Histone modification erase	300,534	XLR	MENTAL RETARDATION, X-LINKED, SYNDROMIC, CLAES-JENSEN TYPE; MRXSCJ
12,637	KDM6A	Histone modification erase	300,867	XLD	KABUKI SYNDROME 2; KABUK2
29,012	KDM6B	Histone modification erase	618,505	AD	NEURODEVELOPMENTAL DISORDER WITH COARSE FACIES AND MILD DISTAL SKELETAL ABNORMALITIES; NEDCFSA
7132	KMT2A	Histone modification write	605,130	AD	WIEDEMANN–STEINER SYNDROME; WDSTS
13,726	KMT2C	Histone modification write	617,768	AD	KLEEFSTRA SYNDROME 2; KLEFS2
7133	KMT2D	Histone modification write	147,920	AD	KABUKI SYNDROME 1; KABUK1
18,541	KMT2E	Histone modification write	618,512	AD	O'DONNELL–LURIA–RODAN SYNDROME; ODLURO
24,283	KMT5B	Histone modification write	617,788	AD	MENTAL RETARDATION AUTOSOMAL DOMINANT 51; MRD51
25,726	LAS1L	Histone modification write cofactor	309,585	XLR	WILSON–TURNER X-LINKED MENTAL RETARDATION SYNDROME
6518	LBR	Anchors the lamina and the heterochromatin to the inner nuclear membrane	613,471	AD	REYNOLDS SYNDROME
6859	MAP3K7	Histone modification write	157,800	AD	CARDIOSPONDYLOCARPOFACIAL SYNDROME; CSCF
20,444	MBD5	Chromatin remodeling	156,200	AD	MENTAL RETARDATION AUTOSOMAL DOMINANT 1; MRD1
6990	MECP2	Histone modification write cofactor, TF	300,055	XLR	MENTAL RETARDATION, X-LINKED, SYNDROMIC 13; MRXS13
6990	MECP2	Histone modification write cofactor, TF	300,260	XLR	LUBS X-LINKED MENTAL RETARDATION SYNDROME; MRXSL. (Alternative name: MECP2 DUPLICATION SYNDROME)
6990	MECP2	Histone modification write cofactor, TF	312,750	XLD	RETT SYNDROME; RTT
7010	MEN1	Histone modification write cofactor	131,100	AD	WERMER SYNDROME (Alternative name: MEN1 syndrome)
7329	MSH6	Histone modification read	619,097	AR	MISMATCH REPAIR CANCER SYNDROME 3; MMRCS3
7370	MSL3	Histone modification read	301,032	XLD	BASILICATA–AKHTAR SYNDROME
29,401	MYSM1	Histone modification erase	618,116	AR	BONE MARROW FAILURE SYNDROME 4; BMFS4
7652	NBN	Chromatin remodeling	251,260	AR	NIJMEGEN BREAKAGE SYNDROME; NBS
18,591	NEK9	Histone modification write	617,022	AR	LETHAL CONGENITAL CONTRACTURE SYNDROME 10; LCCS10
28,862	NIPBL	Histone modification erase cofactor	122,470	AD	CORNELIA DE LANGE SYNDROME 1; CDLS1
14,234	NSD1	Histone modification write	117,550	AD	SOTOS SYNDROME 1
12,766	NSD2	Histone modification write	194,190	AD	WOLF–HIRSCHHORN SYNDROME; WHS
8127	OGT	Histone modification write	300,997	XLR	X-LINKED INTELLECTUAL DEVELOPMENTAL DISORDER 106; XLID106
18,337	PADI3	Histone modification	191,480	AR	UNCOMBABLE HAIR SYNDROME 1; UHS1
12,929	PCGF2	Polycomb group (PcG) protein	618,371	AD	TURNPENNY–FRY SYNDROME; TPFS
8729	PCNA	Chromatin remodeling	615,919	AR	ATAXIA-TELANGIECTASIA-LIKE DISORDER 2; ATLD2
24,156	PHF21A	Histone modification erase cofactor	618,725	AD	INTELLECTUAL DEVELOPMENTAL DISORDER WITH BEHAVIORAL ABNORMALITIES AND CRANIOFACIAL DYSMORPHISM WITH OR WITHOUT SEIZURES; IDDBCS
20,672	PHF8	Histone modification erase	300,263	XLR	MENTAL RETARDATION, X-LINKED, SYNDROMIC, SIDERIUS TYPE; MRXSSD
15,673	PHIP	Histone modification read	617,991	AD	CHUNG–JANSEN SYNDROME; CHUJANS
18,801	POGZ	Histone modification read	616,364	AD	WHITE–SUTTON SYNDROME; WHSUS
9299	PPP2CA	Histone modification write	618,354	AD	NEURODEVELOPMENTAL DISORDER AND LANGUAGE DELAY WITH OR WITHOUT STRUCTURAL BRAIN ABNORMALITIES; NEDLBA
9386	PRKAG2	Histone modification write cofactor	194,200	AD	WOLFF–PARKINSON–WHITE SYNDROME
9399	PRKCD	Histone modification	615,559	AR	AUTOIMMUNE LYMPHOPROLIFERATIVE SYNDROME, TYPE III; ALPS3
25,557	PRMT7	Histone modification write	617,157	AR	SHORT STATURE, BRACHYDACTYLY, IMPAIRED INTELLECTUAL DEVELOPMENT, AND SEIZURES; SBIDDS
9817	RAD51	Histone modification erase	617,244	AD	FANCONI ANEMIA, COMPLEMENTATION GROUP R; FANCR
9831	RAG1	Histone modification write	603,554	AR	OMENN SYNDROME
9832	RAG2	Histone modification read	603,554	AR	OMENN SYNDROME
9834	RAI1	Chromatin remodeling	182,290	AD	SMITH–MAGENIS SYNDROME; SMS
13,429	RLIM	Histone modification erase cofactor	300,978	XLR	TONNE–KALSCHEUER SYNDROME; TOKAS
26,661	RNF168	Histone modification write	611,943	AR	RIDDLE SYNDROME; RIDL
10,061	RNF2	Histone modification write	619,460	AD	LUO–SCHOCH–YAMAMOTO SYNDROME; LUSYAM
10,432	RPS6KA3	Histone modification write cofactor	303,600	XLD	COFFIN–LOWRY SYNDROME; CLS
10,432	RPS6KA3	Histone modification write cofactor	300,844	XLD	X-LINKED INTELLECTUAL DEVELOPMENTAL DISORDER 19; XLID19
10,541	SATB1	Chromatin remodeling cofactor	619,229	AD	KOHLSCHUTTER–TONZ SYNDROME-LIKE; KTZSL
10,541	SATB1	Chromatin remodeling cofactor	619,228	AD	DEVELOPMENTAL DELAY WITH DYSMORPHIC FACIES AND DENTAL ANOMALIES; DEFDA
21,637	SATB2	Chromatin remodeling cofactor	612,313	AD	GLASS SYNDROME
10,760	SET	Histone modification	618,106	AD	MENTAL RETARDATION AUTOSOMAL DOMINANT 58; MRD58
29,010	SETD1A	Histone modification write	618,832	AD	EPILEPSY, EARLY-ONSET, WITH OR WITHOUT DEVELOPMENTAL DELAY; EPEDD
29,010	SETD1A	Histone modification write	619,056	AD	NEURODEVELOPMENTAL DISORDER WITH SPEECH IMPAIRMENT AND DYSMORPHIC FACIES; NEDSID
29,187	SETD1B	Histone modification write	619,000	AD	INTELLECTUAL DEVELOPMENTAL DISORDER WITH SEIZURES AND LANGUAGE DELAY; IDDSELD
18,420	SETD2	Histone modification write	616,831	AD	LUSCAN–LUMISH SYNDROME; LLS
25,566	SETD5	Histone modification write	615,761	AD	MENTAL RETARDATION AUTOSOMAL DOMINANT 23; MRD23
19,353	SIN3A	Histone modification erase cofactor, TF	613,406	AD	WITTEVEEN–KOLK SYNDROME; WITKOS
11,098	SMARCA2	Histone modification read, TF	601,358	AD	NICOLAIDES–BARAITSER SYNDROME; NCBRS
11,098	SMARCA2	Histone modification read, TF	619,293	AD	BLEPHAROPHIMOSIS-IMPAIRED INTELLECTUAL DEVELOPMENT SYNDROME; BIS
11,100	SMARCA4	Histone modification read, TF	613,325	AD	RHABDOID TUMOR PREDISPOSITION SYNDROME 2; RTPS2
11,100	SMARCA4	Histone modification read, TF	614,609	AD	COFFIN-SIRIS SYNDROME 4; CSS4
18,398	SMARCAD1	Chromatin remodeling	129,200	AD	BASAN SYNDROME
18,398	SMARCAD1	Chromatin remodeling	181,600	AD	HURIEZ SYNDROME; HRZ
11,103	SMARCB1	Histone modification read	609,322	AD	RHABDOID TUMOR PREDISPOSITION SYNDROME 1; RTPS1
11,103	SMARCB1	Histone modification read	614,608	AD	COFFIN–SIRIS SYNDROME 3; CSS3
11,105	SMARCC2	Chromatin remodeling cofactor	618,362	AD	COFFIN–SIRIS SYNDROME 8; CSS8
11,106	SMARCD1	Chromatin remodeling	618,779	AD	COFFIN–SIRIS SYNDROME 11; CSS11
11,109	SMARCE1	Chromatin remodeling cofactor	616,938	AD	COFFIN–SIRIS SYNDROME 5; CSS5
11,094	SNAI2	Histone modification erase cofactor	608,890	AR	WAARDENBURG SYNDROME, TYPE 2D
11,254	SPOP	Histone modification write	618,828	AD	NABAIS SA–DE VRIES SYNDROME, TYPE 1; NSDVS1
11,254	SPOP	Histone modification write	618,829	AD	NABAIS SA–DE VRIES SYNDROME, TYPE 2; NSDVS2
16,974	SRCAP	Chromatin remodeling, Histone modification erase	619,595	AD	DEVELOPMENTAL DELAY, HYPOTONIA, MUSCULOSKELETAL DEFECTS, AND BEHAVIORAL ABNORMALITIES; DEHMBA
16,974	SRCAP	Chromatin remodeling, Histone modification erase	136,140	AD	FLOATING-HARBOR SYNDROME; FLHS
11,465	SUPT16H	Histone modification read	619,480	AD	NEURODEVELOPMENTAL DISORDER WITH DYSMORPHIC FACIES AND THIN CORPUS CALLOSUM; NEDDFAC
17,101	SUZ12	Histone modification write cofactor, Polycomb group (PcG) protein, TF	618,786	AD	IMAGAWA–MATSUMOTO SYNDROME; IMMAS
11,535	TAF1	Histone modification write	300,966	XLR	MENTAL RETARDATION, X-LINKED, SYNDROMIC 33; MRXS33
11,535	TAF1	Histone modification write	314,250	XLR	X-linked torsion dystonia-parkinsonism syndrome. (Alternative name: Lubag Syndrome)
11,536	TAF2	Part of novel TFTC-HAT complex, TF	615,599	AR	MENTAL RETARDATION AUTOSOMAL RECESSIVE 40; MRT40
11,540	TAF6	Histone chaperone	617,126	AR	ALAZAMI–YUAN SYNDROME; ALYUS
29,529	TBL1XR1	Targets NCoR repressive complex to deacetylated histones	602,342	AD	PIERPONT SYNDROME; PRPTS
29,529	TBL1XR1	Targets NCoR repressive complex to deacetylated histones	616,944	AD	MENTAL RETARDATION AUTOSOMAL DOMINANT 41; MRD41
28,313	TET3	DNA modification	618,798	AD, AR	BECK–FAHRNER SYNDROME; BEFAHRS
11,842	TLK2	Histone modification write	618,050	AD	MENTAL RETARDATION AUTOSOMAL DOMINANT 57; MRD57
11,998	TP53	Histone modification write cofactor, TF	151,623	AD	LI–FRAUMENI SYNDROME; LFS
11,998	TP53	Histone modification write cofactor, TF	618,165	AD	BONE MARROW FAILURE SYNDROME 5; BMFS5
12,347	TRRAP	Histone modification write cofactor	618,454	AD	DEVELOPMENTAL DELAY WITH OR WITHOUT DYSMORPHIC FACIES AND AUTISM; DEDDFA
12,472	UBE2A	Histone modification write	300,860	XLR	MENTAL RETARDATION, X-LINKED, SYNDROMIC, NASCIMENTO TYPE; MRXSN
12,630	USP7	Histone modification erase, DNA modification cofactor	616,863	AD	HAO–FOUNTAIN SYNDROME; HAFOUS
12,679	VDR	Chromatin remodeling cofactor, TF	277,440	AR	RICKETS–ALOPECIA SYNDROME
12,718	VRK1	Histone modification write	607,596	AR	PONTOCEREBELLAR HYPOPLASIA, TYPE 1A; PCH1A
17,327	WAC	Histone modification write cofactor	616,708	AD	DESANTO–SHINAWI SYNDROME; DESSH
12,856	YY1	Chromatin remodeling cofactor, TF	617,557	AD	GABRIELE–DE VRIES SYNDROME; GADEVS
16,966	ZMYND11	Histone modification read	616,083	AD	MENTAL RETARDATION AUTOSOMAL DOMINANT 30; MRD30
13,128	ZNF711	Histone modification erase cofactor	300,803	XLR	X-LINKED INTELLECTUAL DEVELOPMENTAL DISORDER 97; XLID97

*AD* autosomal dominant, *AR* autosomal recessive, *XLD* X-linked dominant, *XLR* X-linked dominant, *TF* transcription factor

## The OMICs cascade to study pathogenic mutations driving chromatinopathies

**Fig. 2:**
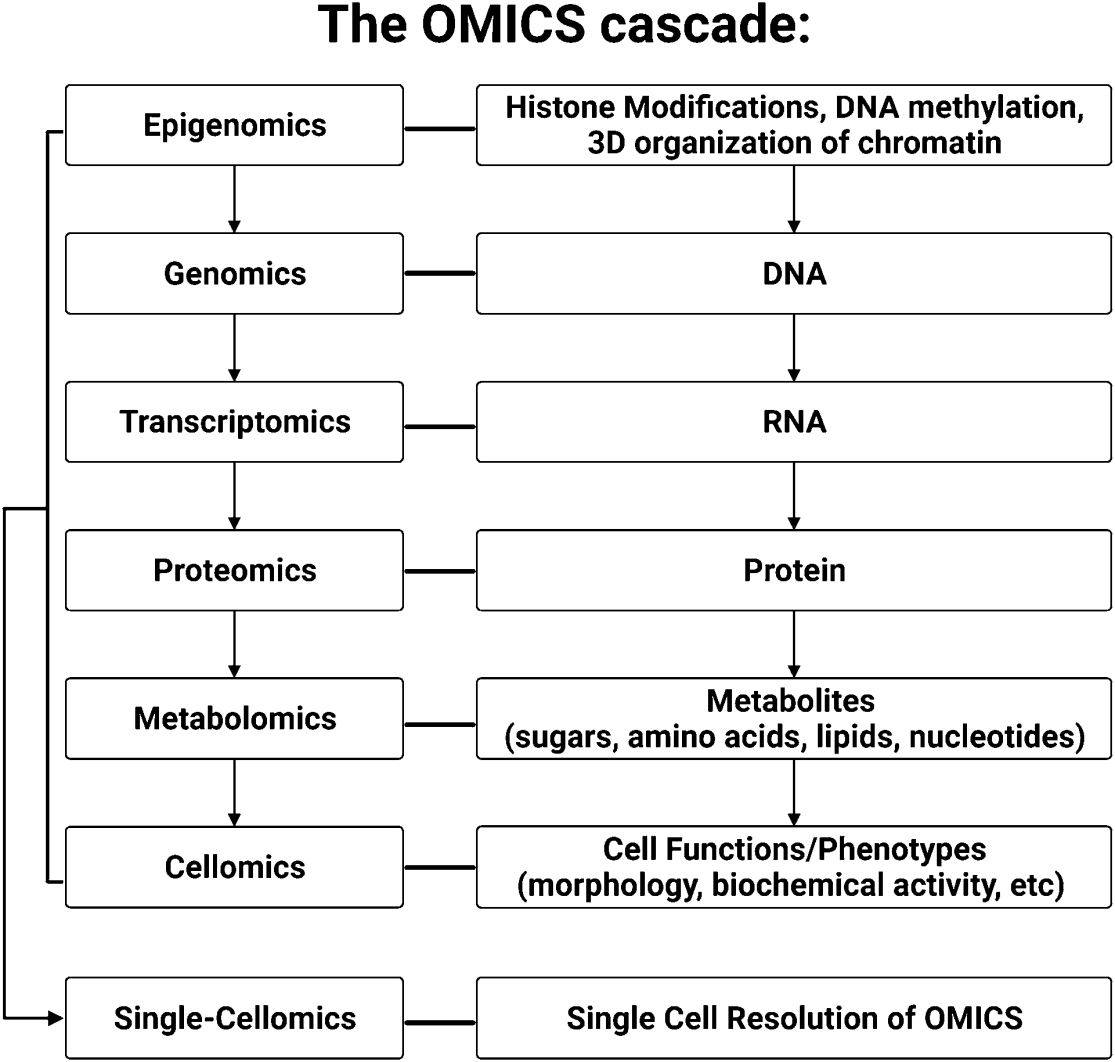
Graphical overview of the OMICs Cascade

The suffix -OMICs is appended to a given field of biology to denote use of high-throughput and high-resolution technologies (Veenstra 2021). Genetic information flows through a 5-layer, hierarchical biological system where each OMICs layer can influence or be influenced by adjacent layers, and all layers can all be assessed at single-cell resolution, referred to here as the “OMICs cascade” (Dettmer et al. 2007) As shown in Fig. 2, each layer of the OMICs cascade highlights a unique biochemical snapshot of a biological system (e.g., cell, tissue, organ, or organism).

The flow of biological information through the -OMICs cascade starts at the epigenome which controls specific activation of cellular programs through chemical modifications on nucleic acids and histones that drive transcription of DNA into RNA. The transcriptome is composed of all the RNA molecules in a cell that are either translated into protein by ribosomes or remain untranslated and function as non-coding RNAs (ncRNAs; e.g., microRNAs, small interfering RNAs, and long ncRNAs). These ncRNA indirectly or directly regulate the expression of their chosen targets through mechanisms such as altered transcript stability (Beermann et al. 2016; Roundtree et al. 2017). The proteome that is encoded by mRNA consists of all the proteins in a biological system (Wilkins 1994) and orchestrates an array of biological processes from cellular homeostasis via ion channels gradients to highly specialized tasks like cell-to-cell communication (Wilkins 2009). Finally, as we move beyond the central dogma of biology, we can assess the metabolome, which is defined as the low molecular weight molecules (i.e., metabolites) present in a biological system that participate in or are a product of biochemical reactions. Metabolites are required for a cell’s normal function, growth, and maintenance (Mosleth et al. 2020; Oliver et al. 1998). The integration of extrinsic stimuli with intrinsic cellular data culminates in cellular phenotypes, termed the cellome (Taylor 2007; Rosato et al. 2021) that are ever expanding with advancements in robotics and imaging capabilities.

The interconnected nature of each OMICs layer enables propagation of perturbations through a biological system. While essential biological processes have developed redundancies to buffer the impact of strong environmental insults, cellular responses are not adapted to respond to exceedingly rare, high effect genetic mutations. Therefore, these rare epigene mutations overwhelm a cell’s buffering capacity, resulting in clinically significant phenotypes or non-viability. Often, a single heterozygous mutation (i.e., one mutated allele and one normal allele) can disrupt multiple cell types and tissues by aberrant activation or repression of signaling pathways, resulting in congenital syndromes (Lin et al. 2022). For example, mutations in the epigene, *CREBBP* cause Rubinstein–Taybi Syndrome 1 (RSTS1; MIM180849) and is a histone acetyltransferase. The -OMICs cascade can be assessed in samples harboring *CREBBP* mutations to assess the cascading effect of the genetic mutation on the epigenome as well as studies of the transcriptome, proteome, and metabolome. Together, these lead to an organismal phenotype seen in the *RSTS1* patients and *Crebbp* knockout mouse models on learning and memory (Lipinski et al. 2022). Targeted studies highlight how aberrant histone acetylation can disrupt multiple layers of molecular and cellular phenotypes. To bridge this gap in knowledge, genome-wide studies of comprehensive OMICs cascade in human and model organisms harboring pathogenic epigene mutations are critical first steps. With multiple epigenes, cell types, and conditions, there are thousands of independent experiments needed to dissect out these complex interplay of the histone code.

### Introduction to performing multi-omic studies on chromatinopathy-related specimens

The dissection of OMICs layers across multiple cell and tissue types can unravel molecular mechanisms driving clinical phenotypes in chromatinopathy patients. Epigenes function at the epigenomic layer at the top of the OMICs cascade. Therefore pathogenic germline mutations in epigenes result in a hierarchical cascading effect through four downstream OMICs layers. The coordinated biochemical disturbances across multiple OMICs layers provide clues about disease pathophysiology and can guide improved diagnostics and therapeutics for the disease. In the following section, we review key examples of the multiple experimental tools (Table 2) that can be used to assay each OMICs layer in chromatinopathies.

**Table 2:** Summary of OMICs techniques

OMICs layer	Molecular aspect assayed	Name of assay
Genomics	DNA sequence	Sanger Sequencing (Sanger et al. 1977) Whole Genome Sequencing (WGS) (Lionel et al. 2018) Whole Exome Sequencing (WES) (Lee et al. 2014) Microarray-based Genotyping
Epigenomics	DNA methylation	Methylation Microarrays(Chater-Diehl et al. 2021), Reduced Representation Bisulfite Sequencing (RRBS) (Meissner et al. 2008), Whole Genome Bisulfite Sequencing (WGBS) (Olova et al. 2018), Methyl Cytosine sequencing (MethylC-seq) (Lister et al. 2008), Methyl DNA ImmunoPrecipitation analyzed by sequencing (MeDIP-seq) (Down et al. 2008), Methyl-CpG Binding Domain-isolated genomic DNA analyzed by sequencing (MBD-seq) (Serre et al. 2010)
	Genomic coordinates of Histone Post-Translational Modifications or Chromatin-associated proteins	Chromatin ImmunoPrecipitation and Sequencing (ChIP-seq) (Johnson et al. 2007)
	Chromatin Accessibility	DNase-seq (Crawford et al. 2006), Assay for Transposase-Accessible Chromatin with Sequencing (ATAC-seq) (Buenrostro et al. 2013), Formaldehyde-Assisted Isolation of Regulatory Elements and Sequencing (FAIRE-seq) (Giresi et al. 2007), MNase-seq (Chereji et al. 2019)
	Chromatin Conformation	Hi-C(Lieberman-Aiden et al. 2009)
	Chromatin Conformation specific for Chromatin-associated proteins	Chromatin Interaction Analysis with Paired-End-Tag sequencing (ChIA-PET) (Fullwood et al. 2009)
	Genomic coordinates of Histone Post-Translational Modifications or Chromatin-associated proteins	Cleavage Under Targets and Release Using Nuclease (CUT&RUN) (Skene and Henikoff 2017), Cleavage Under Targets and Tagmentation (CUT&Tag) (Kaya-Okur et al. 2019)
Transcriptomics	RNA sequence	Short-read RNA sequencing (Lowe et al. 2017), Pacbio's Long-read Isoform sequencing (ISO-seq) (Leung et al. 2021), Oxford Nanopore's Long-read Sequencing (Wang et al. 2021)
Proteomics	Proteins	Western Blots (Pillai-Kastoori et al. 2020), Flow cytometry(Bendall et al. 2012), Mass Spectrometry (MS) (Yates et al. 2009), Multiplexed ImmunoHistoChemistry (IHC) / ImmunoFluorescence (IF) (Tan et al. 2020), Protein Microarrays(Chandra et al. 2011), SOMAscan, a High-throughput proteomics platform(Kim et al. 2018), Proximity Ligation Assay (PLA) (Weibrecht et al. 2010), Proximity Extension Assay (PEA) (Assarsson et al. 2014)
Metabolomics	Metabolites	Mass Spectrometry (MS) (Perez-Ramirez and Christofk 2021), Nuclear Magnetic Resonance (NMR) (Perez-Ramirez and Christofk 2021), Biochemical assays(Perez-Ramirez and Christofk 2021), Image-based technologies (Perez-Ramirez and Christofk 2021), Cellomics Cellinsight High Content Screening Platform (Ardashov et al. 2019), Cellomics ArrayScan platform (Williams et al. 2006), Opera™ LX (PerkinElmer) automated confocal microscopy system (Rosato et al. 2021)

*Websites Accessed* https://epifactors.autosome.org/, version 1.7.3

A successful multi-omics study design in human specimens can be achieved using multiple strategies and cell types. Assessing an epigene’s RNA and protein expression profile can identify which cell- or tissue-type(s) will yield the most meaningful results. In the context of Mendelian Syndromes, this type of multi-tissue sampling strategy can identify pathogenic mechanisms that remain constant across multiple cellular contexts (Lin et al. 2022; Götz et al. 2008). Furthermore, a multi-omics approach can identify which cells and tissues are particularly vulnerable or resilient to disruption of a specific epigene. In some cases, sampling of most appropriate cells or tissues is not possible, as there are ethical limitations or impossible to obtain. Therefore, in vitro modeling of specific cell types using stem cells is an attractive and highly relevant alternative approach. For chromatinopathy syndromes, many of the epigenes are highly expressed in early mammalian embryonic development (Nestorov et al. 2015), and functional studies in model organisms have shown that they are critically important in regulating stem cell pluripotency and differentiation (Katsumoto et al. 2006; Gan et al. 2007; Alari et al. 2018).

To assay the tissue-specific effects of pathogenic epigene mutations with the -omics techniques listed in Table 2, we can use human induced pluripotent stem cells (iPSCs) harboring patient-specific mutations or artificially created using gene editing. Since iPSCs have the potential to differentiate into all three germ layers (endoderm, ectoderm, and mesoderm) and all somatic cell types, they enable the in vitro recapitulation of early developmental in vivo events (Tiscornia et al. 2011; Loh et al. 2014, 2016; Tchieu et al. 2017; Durbin et al. 2018; Rowe and Daley 2019). iPSC models allow researchers to investigate disease-associated mechanisms in a temporal- and cell-type specific manner (Matheus et al. 2019; Carosso et al. 2019; Calzari et al. 2020). While iPSC-derived cells allow study of unobtainable cell types, it is known that stem cell studies suffer from problems with reproducibility that can be caused by: technical variability, genetic heterogeneity, and biological variation (Volpato and Webber 2020). However, the stem cell field is actively devising guidelines and testing methodologies to improve reproducibility as iPSCs are invaluable for in vitro disease modeling (Volpato et al. 2018; Anderson et al. 2021; Reed et al. 2021; Birbrair 2021; Brunner et al. 2022).

Performing these experiments across all epigenes, cell types and experimental conditions would cost billions of dollars and therefore creative methods for combining samples and decreasing sample requirements can improve our ability to comprehensively study the role of the epigenome in human disease. Despite the potential roadblocks to high-quality multi-omics studies, we believe assaying even a subset of cell types across the mutational spectrum will identify targetable and novel pathogenic mechanisms, potential disease-modifying gene networks, and diagnostic and monitoring biomarkers (Awamleh et al. 2022) for use in clinical trials.

For the following OMICs subsections, we first briefly introduce technologies that are commonly used to assay a given layer, we then highlight salient examples where these OMICs technologies were applied to chromatinopathy-related biological specimens such that novel disease-associated properties were identified. We highlight the fact that of the 179 chromatinopathies identified in this review (Table 1), only six chromatinopathies (i.e., Kabuki Syndrome 1 and 2, Rubinstein–Taybi Syndrome 1 and 2, Rett Syndrome, and Bohring Opitz Syndrome) have been thoroughly studied using a multi-omics approach in disease-relevant cell types (Berdasco and Esteller 2013; Bjornsson 2015; Fallah et al. 2020; Fahrner and Bjornsson 2014; Faundes et al. 2018; Lin et al. 2022). There remains a huge potential for major discoveries in the chromatinopathy field that will lead to the development of novel therapeutics.

### Epigenomics

Each aspect of the epigenome can be precisely measured using high-throughput techniques to understand how the epigenome changes across biological contexts (Mehrmohamadi et al. 2021). The most progress has been made in developing DNA methylation-based epi-signatures, which capture the DNA methylation changes caused by a pathogenic mutation that can then be used to distinguish genetic variants of uncertain significance as benign or pathogenic (Chater-Diehl et al. 2021; Awamleh et al. 2022). These tools can be used as a next-line test to end the diagnostic odyssey by classifying a variant as causal for the syndrome or as a benign variant. Another use of epigenetic biomarkers is for therapeutic monitoring to determine whether precision targeted treatments drugs can reverse the effect of pathogenic mutation on the DNA methylation episignature (Butcher et al. 2017; Awamleh et al. 2022). To generate DNA methylation episignatures, patient DNA undergoes bisulfite chemical conversion (Fig. 1) and then is profiled on a methylation array containing 850,000 CpG methylation sites or by sequencing (Pidsley et al. 2016). A recent paper demonstrated that *ASXL1* mutations that cause Bohring–Opitz Syndrome (BOS) have a distinct methylation episignature from other chromatinopathy disorders, like Kabuki syndrome, Sotos syndrome, and Weaver syndrome (Awamleh et al. 2022). Specifically, 763 differentially methylated CpG sites in BOS patients were used to develop the episignature and these classified variants of unknown significance (VUS) in *ASXL1* by combining machine learning with the BOS episignature—thereby expanding the diagnostic tools available for this chromatinopathy (Awamleh et al. 2022). In a separate study, researchers derived methylation signatures from patients with 50 different chromatinopathies and created a Methylation Variant Pathogenicity (MVP) score which quantifies the probability that a score matches a specific disease (Sadikovic et al. 2021). One major challenge in rare disease studies is the need for robust replication and reproducibility of biomarkers. The standard in the field is to provide the basic summary of which methylation sites were used to generate the episignatures (Choufani et al. 2020). However, availability of raw data would provide immense benefit to the rare disease community. To date, many studies fail to provide raw or summary data which prevent validation in other data sets and reproducibility (Levy et al. 2022).

### Genomics

Pathogenic mutations in epigenes that occur in the germline leads to Chromatinopathies and mutations that arise in somatic cells lead to cancer development. (Berdasco and Esteller 2013; Fahrner and Bjornsson 2014; Bjornsson 2015; Wainwright and Scaffidi 2017; French and Pauklin 2021). Cataloging common mechanisms caused by epigene mutations across disease can point toward precision therapies for both types of disorders (Russell et al. 2015; Slatnick et al. 2023). Investigating the specific epigene mutations that cause existing chromatinopathies remains critical as mutations within several epigenes (e.g., *CREBBP*, *EP300*, *KAT6B*, *DNMT3A*) cause more than one developmental syndrome with no established mechanism for the distinct clinical presentations. For example, mutations predicted to cause premature truncation variants in *KAT6B* cause two recognized syndromes: Genitopatellar Syndrome (GPS) (Campeau et al. 2012) and Say–Barber–Biesecker–Young–Simpson Syndrome (SBBYSS) (Clayton-Smith et al. 2011). However, a recent study highlighted a significant overlap and presence of an intermediate clinical phenotype with features of both GPS or SBBYSS (Zhang et al. 2020) and that these differences may be due to the variable location of the pathogenic mutation within the gene body of *KAT6B (*Yabumoto et al. 2021*)*. The paralog of *KAT6B*, which is *KAT6A*, causes a single chromatinopathy called Arboleda–Tham Syndrome (ARTHS) and patients display phenotypic variability that is correlated with location of the mutation within the gene body of *KAT6A* (Kennedy et al. 2018). Understanding how truncations affect gene and protein function can influence response to precision therapies, when they become available. A clear understanding how specific mutations drive different causal mechanisms and clinical phenotypes will be essential to determining whether therapies will be equally effective across all mutations observed in patients.

### Transcriptomics

The ability to vary exon usage in a transcript exponentially increases the diversity of RNA isoforms possible within a cell and ultimately drives the protein diversity. Many genes expressing multiple isoforms per cell type (Djebali et al. 2012). Pathogenic epigene mutations can disrupt gene expression, splicing, alternative polyadenylation, and accessibility of transcriptional start sites which leads to disease phenotypes. RNA sequencing technologies (Bolisetty et al. 2015; Jeffries et al. 2020) allows study of isoforms-specific effects of epigene mutations that translate across cell and developmental time. These studies have the power to inform the effect of genomic variants that fall outside of the canonical protein-coding regions and affect splice isoforms.

Recently, the clinical utilities of transcriptome studies have been used to functionally validate rare pathogenic splice variants that disrupt genes causing rare Mendelian Disease (Cummings et al. 2017; Lee et al. 2020). Transcriptomic analysis can also reveal isoform-specific pathogenic mechanisms underlying chromatinopathy syndromes. In Rett syndrome, an X-linked chromatinopathy caused by heterozygous mutations in the gene *MECP2*, researchers discovered alternative splicing of the *MECP2* transcript led to the production of a novel isoforms with different N-terminus relative to the canonical *MECP2* transcript (Kriaucionis and Bird 2004; Mnatzakanian et al. 2004). Specifically, at the time, the canonical *MECP2* transcript included exons 1 through 4 and translation of this isoform began at the “ATG” present in exon 2 (*MECP2e2)*—while the newly discovered *MECP2* transcript excluded exon 2 via alternative splicing to generate a novel isoform whose translation begins at the “ATG” present in exon 1 (*MECP2e1) (*Kriaucionis and Bird 2004; Mnatzakanian et al. 2004*)*. A subset of Rett syndrome patients had mutations affecting only MECP*2e1*—suggesting that the exon1 ATG isoform was the critical isoform leading to Rett Syndrome (Djuric et al. 2015). iPSCs carrying a *MECP2e1*-specific mutation (Djuric et al. 2015) caused reduced neuron soma size and altered synaptic activity compared to controls (Djuric et al. 2015). Exogenous expression of wild-type *MECP2e1*, but not wild-type *MECP2e2*, resulted in the phenotypic rescue of neuron cell-body size (Djuric et al. 2015).

### Proteomics

The human proteome represents the functional biological machinery and is the primary target for disease-modifying therapies. Protein abundance is regulated by the rates of translation and degradation, and protein function and stability is mediated by post-translational modifications. Mutations in epigenes are most frequently considered to disrupt the ability to identify, add, or remove post-translational modifications from histone marks (Aebersold and Mann 2016; Li et al. 2021). The workhorse machine driving proteomics-based discovery is the mass spectrometer (MS) which leverages differences in peptide mass-to-charge ratios to identify thousands of proteins and hundreds of protein post-translational modifications (PTMs) in tandem (Witze et al. 2007; Bantscheff et al. 2012; Silva et al. 2013). In the context of human disease, MS-based techniques are mainly used to quantify relative or absolute differences in peptide abundance across affected and unaffected individuals to pinpoint disease-specific proteomic changes (Altelaar et al. 2013). Importantly, these disease-specific proteomic changes can be used as biomarkers in the clinical diagnosis and treatment of various human morbidities, ranging from genetic disorders to infectious diseases and cancers (Fleurbaaij et al. 2015; Diedrich and Dengjel 2017; Daniel and Turner 2018; Chapman and Thoren 2020; Pančík et al. 2022; Wang et al. 2022). Since the epigenome has been implicated in various human morbidities and histone PTMs play a pivotal role in modulating the epigenome (Figs. 1, 2), it is no surprise that histone PTMs are being profiled to understand disease pathophysiology (Thygesen et al. 2018; Cobos et al. 2019; Azevedo et al. 2022; Lempiäinen and Garcia 2023).

In the context of chromatinopathies, a majority of the proteomic data that exists from patient-derived biological specimens (i.e., plasma, fibroblasts, iPSC-derived lineages) pertains to Rett syndrome (Cortelazzo et al. 2013; Pecorelli et al. 2016; Kim et al. 2019; Varderidou-Minasian et al. 2020; Cicaloni et al. 2020b, a). In an unbiased proteomic approach using label-based MS, researchers found that neural lineages generated from Rett syndrome iPSCs showed aberrant protein expression in genes related to differentiation (Kim et al. 2019). In this time-course study, they performed MS on Rett syndrome and control iPSC-derived neural progenitor cells (NPCs) and neural cultures (Kim et al. 2019). Their proteomic analyses revealed NPCs derived from Rett syndrome patients displayed significantly reduced glial fate (GFAP +) and increased neuronal fate (MAP2 +) after three weeks of differentiation (Kim et al. 2019). Moreover, they found the suppression of glial fate in *MECP2* mutant NPCs (i.e., those from Rett syndrome iPSCs) is due to overexpression of LIN28, a RNA binding protein that had been previously shown to blocks the differentiation into glia and increases differentiation into neurons (Balzer et al. 2010). The multi-faceted proteomics data suggest that Rett syndrome’s neuropathology is due to a cell-fate timing defect in early brain development. This study demonstrates proteomic approaches can uncover potential disease-causing mechanisms and underscores the importance of studying chromatinopathies in disease-relevant cell types at various points across developmental time.

### Metabolomics

The metabolome is made up of low molecular weight metabolites, such as sugars, amino acids, lipids, and nucleotides (Dettmer et al. 2007), many of which are used in post-translational histone modifications that are important for writing the ‘histone code’ (Fig. 1) (Cheng and Kurdistani 2022; Hsieh et al. 2022). Metabolic phenotyping across samples with epigene mutations can identify novel biomarkers for disease (Remmel et al. 2016; Dettmer et al. 2007; Nicholson et al. 2012; Justice et al. 2013) due to the build-up of certain metabolic by products (Moser et al. 2007) and also serve as a marker as to whether a given treatment is having an effect. The metabolome of cells can be measured both quantitatively and qualitatively using various techniques that can be divided into four general categories: MS, nuclear magnetic resonance, biochemical assays/panels, and imaging-based analyses (Lu et al. 2017; Perez-Ramirez and Christofk 2021). However, the most common metabolomic approach is to assay metabolites in biological specimens using LC–MS/MS which couples liquid with dual mass spectrophotometry detectors for enhanced coverage of metabolites. As of 2022, ~ 253,000 metabolites and their reference spectra have been cataloged in The Human Metabolome Database (HMDB) which contains 61 different types of biological specimens (Wishart et al. 2022), understanding the cause-and-effect driving metabolic changes in patients with epigene mutations is vital to developing therapeutics for these disorders.

Most of the existing metabolomics data generated from chromatinopathy biological specimens relate to the study of Rett syndrome (Pecorelli et al. 2016; Cappuccio et al. 2019; Neul et al. 2020), Rubinstein–Taybi syndrome 1 and 2 (Welters et al. 2019), and Kabuki syndrome (Pacelli et al. 2020). The first publication to identify a metabolic defect in Rett syndrome found Rett syndrome patients had high lipid levels (i.e., total cholesterol, LDL cholesterol, and HDL cholesterol) (Sticozzi et al. 2013). In Rett syndrome fibroblasts, the hyperlipidemia is caused by altered PTM of SRB1, which encodes a receptor modulating cholesterol trafficking (Shen et al. 2018). A third independent study used MS to analyze over 900 plasma metabolites in Rett syndrome patients (Cappuccio et al. 2019). Pathway-based analysis for Rett syndrome dysregulated metabolites identified sphingolipid metabolism as a core pathway (Cappuccio et al. 2019). Taken together, these three independent metabolomic studies corroborated the hypothesis that lipid dysregulation is a key feature in Rett syndrome. These studies serve as a potential framework for other chromatinopathies that have metabolic disease-associated phenotypes.

### Cellomics

The biological information from the upstream OMICs layers is integrated into a unique molecular state that produces a cellular phenotype, termed cellome. The cellome is traditionally assayed using high-content screens that capture cell properties, such as proliferation, size, migration (Matheus et al. 2019), morphology (Rosato et al. 2021), signaling (Gierisch et al. 2020), cell death, cell cycle, and organelle morphology (Iannetti et al. 2019) and density (Dawes et al. 2007; Taylor 2007). These dynamic cell properties are can be quantified using high-throughput fluorescent microscopy, flow cytometry, and plate readers and more automated systems quantifying specific phenotypes remain to be seen. Using functional assays and fluorescent microscopy, the following example identifies the aberrant cell phenotypes observed in lineages descending from BOS patient iPSCs, thereby expanding our understanding of BOS pathology (Matheus et al. 2019). In the case of rare chromatinopathies, it may not be possible to generate multiple patient iPSC lines. Therefore, genome editing of human pluripotent stem cells (hPSCs) offers an alternative approach increasing the total number of independent biological replicates that can be used to study pathogenic mutations. Matheus et al. used iPSC lines derived from two BOS patients, in conjunction with four biologically-independent *ASXL1* lines that were created via genome editing, to study dosage (heterozygous vs homozygous) and the effect of overexpression of the full-length and truncated mutant. They demonstrated that in all *ASXL1* truncation paradigms, hPSC-derived neural crest (NC) cells showed significantly decreased migration in vitro and in vivo compared to controls (Matheus et al. 2019). Comparing the knockout and overexpression *ASXL1* hPSC-derived NC models, demonstrated that full-length *ASXL1* is required for normal NC migration and that the presence of any truncated *ASXL1* protein is sufficient for perturbation of NC migration. Using disease-relevant cell types, this study identifies aberrant mechanisms that likely underlie the NC-related phenotypes observed in BOS.

## Discussion

This review establishes a broader definition of chromatinopathy-causing epigenes and more than double the number of chromatinopathy syndromes previously reported in the literature (Table 1**)**. The new list includes 720 epigenes with expanded definition of epigene functions. A total of 17 unique functions were described for proteins that directly alter the epigenome: (1) histone “writer”, (2) histone “eraser”, (3) histone “reader”, (4) chromatin “remodeler”, (5) histone chaperone, (6) scaffold protein, (7) DNA modifier, (8) RNA modifier, (9) polycomb group protein, (10) transcription factor, (11) protein cofactor for histone “writer”, (12) protein cofactor for histone “eraser”, (13) protein cofactor for histone “reader”, (14) protein cofactor for chromatin “remodeler”, (15) protein cofactor for histone chaperone, (16) protein cofactor for DNA modifier, and (17) protein cofactor for RNA modifier. Protein cofactors are essential for the optimal activity of complexes formed by epigenes that perform the associated epigenome-related function. A prime example of a protein cofactor is the chromatinopathy-causing epigene *TRRAP*, which is considered a histone “writer” cofactor because it binds to chromatin to recruit histone acetyltransferase complexes to a target sites (Murr et al. 2007; Cogné et al. 2019; Yin and Wang 2021). It is difficult to directly compare our approach to curation used by earlier publications describing chromatinopathy-causing genes due to insufficient description of their curation approach (Berdasco and Esteller 2013; Gabriele et al. 2018; Fahrner and Bjornsson 2019; Wilson et al. 2022; Nothof et al. 2022). Our list of chromatinopathy-causing epigenes (Table 1) creates a valuable resource for the scientific community.

Across the chromatinopathy genes, it is evident we have only scratched the surface of epigene mechanisms in human development and disease. Studies of rare chromatinopathies using patient- biospecimens will be essential to understanding how epigene mutations perturbs essential downstream pathways to cause disease. OMICs studies can link pathogenic mutations with specific biological perturbations and the emerging single-cell approaches will offer improved resolution of the biological changes within a disease state. For example, developing an integrated understanding of the multiple layers of the OMICs cascade can improve our identification of cell-, tissue- and developmentally specific markers. The novel information gained from multi-omic studies can be used to develop diagnostic biomarkers, to discover new chromatinopathies, to identify potential disease-modifying pathways, and pinpoint disease-causing mechanisms. There exist several reviews that cover the logistics of performing multi-omic studies and what computational tools are available for integration of data from multiple OMICs layers (Misra et al. 2018; Subramanian et al. 2020; Hill and Gerner 2021). Finally, to ensure reproducibility, it is imperative that researchers publish detailed information on experimental design, data analysis pipelines and raw data from their large-scale studies (Krassowski et al. 2020). Furthermore, national and global institutions have begun to address the lack of reproducibility by requiring that the raw data be easily accessible to prevent siloing of precious patient-data and fabrication of results. Chromatinopathy disorders are rare and every study, particularly those that use patient-derived samples, is a step toward identifying disease mechanisms and drug targets. With increased sharing of OMICs data derived from chromatinopathy patients, we can make true progress in the diagnosis and treatment of these rare disorders.

## Data Availability

All analyses from this study is available in the supplementary files or main text. We did not generate raw data for this study.
